# Population Pharmacokinetics and Dosing Simulation of Vancomycin Administered by Continuous Injection in Critically Ill Patient

**DOI:** 10.3390/antibiotics10101228

**Published:** 2021-10-09

**Authors:** Romain Garreau, Benoît Falquet, Lisa Mioux, Laurent Bourguignon, Tristan Ferry, Michel Tod, Florent Wallet, Arnaud Friggeri, Jean-Christophe Richard, Sylvain Goutelle

**Affiliations:** 1 Hospices Civils de Lyon, Groupement Hospitalier Nord, Service de Pharmacie, 69005 Lyon, France; romain.garreau@chu-lyon.fr (R.G.); BFalquet@lhopitalnordouest.fr (B.F.); lisa.mioux@chu-lyon.fr (L.M.); laurent.bourguignon@chu-lyon.fr (L.B.); michel.tod@chu-lyon.fr (M.T.); 2 Laboratoire de Biométrie et Biologie Evolutive, Université Lyon 1, UMR CNRS 5558, 69100 Villeurbanne, France; 3 Facultés de Médecine et de Pharmacie de Lyon, Université Lyon 1, 69008 Lyon, France; tristan.ferry@chu-lyon.fr (T.F.); arnaud.friggeri@chu-lyon.fr (A.F.); jean-christrophe.richard@chu-lyon.fr (J.-C.R.); 4 Hospices Civils de Lyon, Groupement Hospitalier Nord, Hôpital de la Croix-Rousse, Service des Maladies Infectieuses et Tropicales, 69004 Lyon, France; 5 Centre Hospitalier Lyon Sud, Hospices Civils de Lyon, Critical Care, 69495 Pierre-Bénite, France; florent.wallet@chu-lyon.fr; 6 Centre International de Recherche en Infectiologie (CIRI) Inserm, Public Health, Epidemiology and Evolutionary Ecology of Infectious Diseases (PHE3ID), U1111, UCBL Lyon 1, CNRS, UMR5308, ENS de Lyon, 69364 Lyon, France; 7 Hospices Civils de Lyon, Groupement Hospitalier Nord, Hôpital de la Croix-Rousse, Service de Médecine Intensive–Réanimation, 69004 Lyon, France

**Keywords:** vancomycin, pharmacokinetics, continuous infusion, ICU

## Abstract

**Background:** Vancomycin is widely used for empirical antimicrobial therapy in critically ill patients with sepsis. Continuous infusion (CI) may provide more stable exposure than intermittent infusion, but optimal dosing remains challenging. The aims of this study were to perform population pharmacokinetic (PK) analysis of vancomycin administered by CI in intensive care unit (ICU) patients to identify optimal dosages. **Methods:** Patients who received vancomycin by CI with at least one measured concentration in our center over 16 months were included, including those under continuous renal replacement therapy (CRRT). Population PK was conducted and external validation of the final model was performed in a dataset from another center. Simulations were conducted with the final model to identify the optimal loading and maintenance doses for various stages of estimated creatinine clearance (CR_CL_) and in patients on CRRT. Target exposure was defined as daily AUC of 400–600 mg·h/L on the second day of therapy (AUC24–48 h). **Results:** A two-compartment model best described the data. Central volume of distribution was allometrically scaled to ideal body weight (IBW), whereas vancomycin clearance was influenced by CRRT and CR_CL_. Simulations performed with the final model suggested a loading dose of 27.5 mg/kg of IBW. The maintenance dose ranged from 17.5 to 30 mg/kg of IBW, depending on renal function. Overall, simulation showed that 55.8% (95% CI; 47–64%) of patients would achieve the target AUC with suggested dosages. **Discussion:** A PK model has been validated for vancomycin administered by CI in ICU patients, including patients under CRRT. Our model-informed precision dosing approach may help for early optimization of vancomycin exposure in such patients.

## 1. Introduction

In critically ill patients, infection due to Gram-positive bacteria remains a major cause of mortality [1]. Vancomycin remains widely used to treat these pathogens, including methicillin-resistant *Staphylococcus aureus* (MRSA) and ampicillin-resistant enterococci [2]. Vancomycin is also widely prescribed as part of broad-spectrum probabilistic antibiotic therapy in critically ill patients with sepsis, which can alter drug disposition and result in inappropriate drug exposure [3,4]. Moreover, the use of continuous renal replacement therapy (CRRT), which is sometimes necessary in septic critically ill patients, may further alter drug pharmacokinetics [5]. The traditional dosing of vancomycin is based on intermittent infusions (II) of 1–2 h. However, intermittent infusion of vancomycin may be inadequate in critically ill patients who require stable drug exposure [6,7]. Indeed, several studies have reported a more stable exposure, higher target attainment, and lower rate of nephrotoxicity in patients under continuous infusion (CI) of vancomycin compared with intermittent infusion [2,4,8,9,10,11,12,13]. The Infectious Disease Society of America (IDSA) has recently updated its guidelines about vancomycin therapeutic drug monitoring [14] (TDM), with the main recommendation being to switch from trough-based TDM to AUC-based TDM, since the latter has been associated with lower rates of nephrotoxicity [15].

In addition to the advantages cited above, CI of vancomycin can facilitate AUC-based therapeutic drug monitoring that is now recommended [16]. Indeed, assuming that steady-state has been achieved, the daily AUC can be directly calculated by multiplying the measured steady-state concentration (Css) by 24. Guidelines have suggested Css target of 20–25 mg/L for CI vancomycin TDM to achieve an AUC target of 400–600 mg·h/L and an AUC/MIC ratio of 400–600, based on a putative MIC of 1 mg/L for MRSA infections [14,17].

However, optimal dosing of vancomycin administered by CI in critically ill patients remains challenging. Clinicians must adjust the initial loading and maintenance doses to the patient’s needs before TDM can be performed and used for subsequent individualization.

While there are some recommendations to use a 20–35 mg/kg loading dose, there is no formal recommendation regarding the maintenance dosing regimen. Some studies have suggested to use a higher dosage of vancomycin in case of continuous infusion [18,19].

Therefore, this study aimed to perform a population pharmacokinetics analysis of vancomycin administered by continuous infusion in critically ill patients, and to determine a dosing regimen to better achieve target exposure over the first 48 h of therapy.

## 2. Results

### 2.1. Patient Characteristics

Patient characteristics recorded on hospital admission (baseline for both populations (learning and validation) are described in Table 1.

In the learning set, 335 vancomycin concentrations were available from 78 patients hospitalized in ICU. The mean TDM follow up time was 4.1 ± 2 days. Mean vancomycin loading and maintenance dosages were 22.7 ± 7.5 mg/kg and 28.6 ± 9.4 mg/kg/day respectively. A total of 22 out of 78 patients (28.2%) of patient were under CRRT

In the validation group, 417 samples were obtained from 84 patients. The mean TDM follow-up time was 5.0 ± 1.9 days. Mean vancomycin loading and maintenance dosages were 22 ± 5.8 mg/kg and 25.2 ± 7.6 mg/kg/day, respectively, and 4 out of 84 patients (4.8%) of patients were under CRRT.

### 2.2. Population PK Modeling

The model that best described the data was a two-compartment model with a proportional residual error. Mean vancomycin clearance (CL), central volume (V1), intercompartment clearance (Q), and peripheral volume (V2) were 0.79 L/h, 27.3 L, 6.1 L/h, and 63.1 L, respectively. All parameters were estimated with an acceptable standard error, as shown in Table 2.

**Figure 1:**
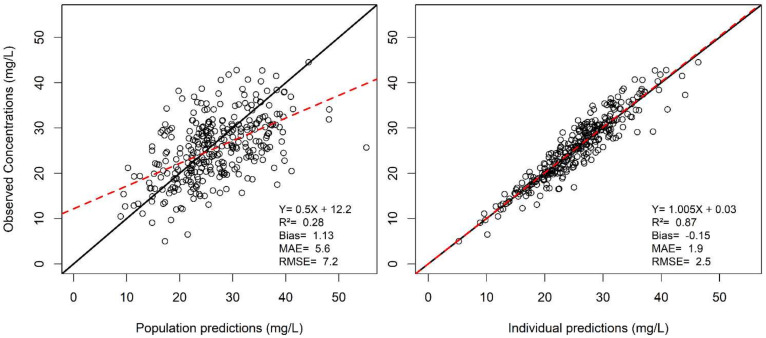
Observed versus predicted vancomycin concentrations in the learning dataset.

Creatinine clearance, IBW, and CRRT_EFR_ were retained as influential covariates in the final model. Vancomycin CL was modeled as a function of CR_CL_ in patients without CRRT, and as a function of CRRT_EFR_ in patients on renal replacement therapy. V1 was allometrically scaled to ideal body weight. Population and individual predictions from the model correlated well with observations, and bias and imprecision were low, as shown in Figure 1.

Observed concentrations (Y axis) versus predicted concentrations (X axis) are shown for population predictions (left panel) and individual predictions (right panel). The black line is the identity line (Y = X) and the red line is the linear regression line. Bias is the mean error of prediction. MAE = mean absolute error and RMSE = root mean squared error.

**Figure 2:**
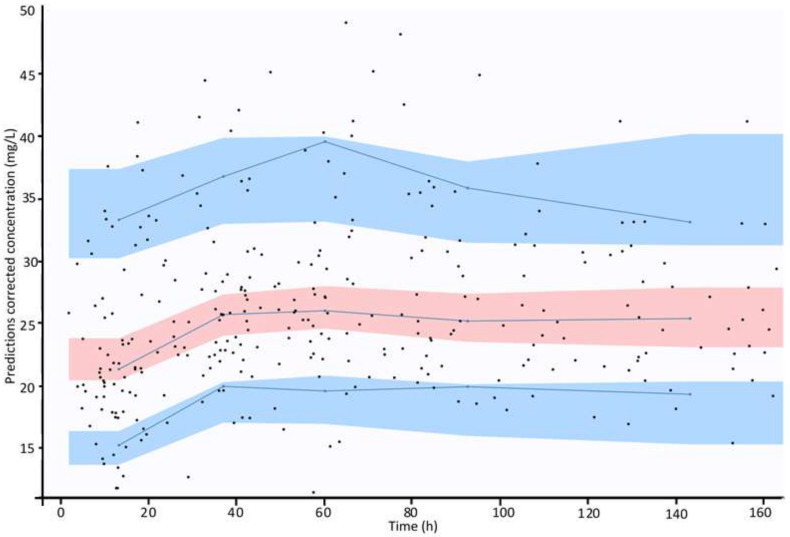
Internal validation of the model: prediction-corrected visual predictive checks.

Figure 2 shows the prediction-corrected visual predictive checks. Percentiles of observations lied entirely within the confidence limits of simulated percentiles, which support internal validation of the model.

Concentrations of vancomycin (Y axis) are plotted against time after the first dose (X axis). Black dots represent observed concentration data. Blue lines represent the 10th, 50th (median) and 90th percentiles of observations. Blue areas represent the 95% prediction interval of the 10th and 90th simulated percentiles, while the magenta area represents the 95% prediction interval of the simulated median.

**Figure 3:**
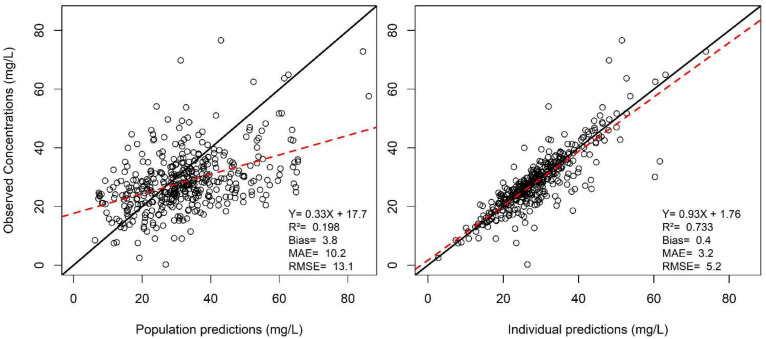
Observed vs. predicted concentration in the validation dataset.

Figure 3 shows the result of the external validation. Model predictions correlated well with observations, with low bias and imprecision Overall, results supported the predictive performance of the model and its use for subsequent dosing simulations.

Observed concentrations (Y axis) versus predicted concentrations (X axis) are shown for population predictions (left panel) and individual predictions (right panel). The black line is the identity line (Y = X), the red line is the linear regression line. Bias is the mean error of prediction. MAE = Mean absolute error, RMSE = Root mean square error

### 2.3. Dosing Simulations

Results of dosing simulations targeting AUC24–48 h between 400 and 600 mg·h/L are summarized in Table 3. The same loading dose of 27.5 mg/kg (IBW) was applied for all stages of renal function. In patients without CRRT, simulated maintenance doses ranged from 17.5 to 30 mg/kg/day (IBW). The median probability of efficacy target attainment (PTA) ranged from 69 to 83%, while the median risk of overexposure ranged from 17.6 to 31%. Overall, 55.8% (95% CI; 47–64.2%) of patients would achieve the AUC target interval on the second day of therapy with the suggested regimens. In patients with CRRT, the maintenance dose varied with CRRT effluent rate.

Results of the second set of simulations targeting AUC_24–48_ > 400 are shown in Table 4. This strategy would be associated with higher dosages, reduction of underexposure, but also higher risk of AUC beyond the upper bound.

## 3. Discussion

This study investigated the population PK of vancomycin administered as continuous infusion in critically ill patients. The final model adequately described the data and was externally validated. Ideal body weight, CR_CL_, and CRRT were found to influence vancomycin PK. Simulations performed with the final model provided initial dosage regimens optimizing achievement of the AUC target in this clinical setting.

Vancomycin remains widely prescribed as a probabilistic antibiotic therapy to treat sepsis in critically ill patients [3]. However, it is a potentially toxic drug [20], especially in ICU patients which may have changing pharmacokinetic parameters [21]. Several pharmacokinetics models have been previously published on vancomycin in ICU patients. However, none of them has been validated for CI, which has been associated with more stable exposure [16,22]. Recently, vancomycin nephrotoxicity has been shown to be associated with supra-therapeutic through concentration and may appear as early as 48 h after therapy onset [23]. Therefore, we decided to create a population PK model to optimize vancomycin AUC while minimizing the risk of adverse events during the first hours of therapy before the first TDM.

In a recent review on vancomycin population pharmacokinetics model [24], the authors reported a mean CL of 0.051 L h kg^−1^ (of TBW) and median volume of distribution of 0.864 L/kg (TBW). Those values are higher than the estimates from our study, with a typical CL of 0.0185 L h^−1^ kg^−1^ (TBW) and a typical V1 of 0.43 L kg^−1^ (TBW). However, we reported a larger coefficient of variation of V1 (63.8%) while the coefficient of variation of CL in our cohort was similar to previous study results [25]. It has been suggested that cardiac output should be explored as a potential covariate that could influence both CL and V. Indeed, a reduced cardiac output could be associated with a change in renal blood flow, influencing renal elimination. Imaura et al. [26] reported a correlation between the central volume and the EVLWI. Variable collected via PICCO monitoring were assessed as covariates, but none showed significance influence. This may be due to the sampling design, as estimation of V1 after the loading dose was based on sparse sampling. Furthermore, during model building, we found an influence of the use of inotropic agents on vancomycin clearance, but it was not retained in the final model because of borderline significance (*p* = 0.067). The effect of inotropic agents on vancomycin PK has been described elsewhere [27].

We found that IBW was a better descriptor of vancomycin V1 compared with TBW. Alternative body size descriptors have been thoroughly studied in pharmacokinetic studies in pediatric and obese adults [28]. However, to our knowledge, no study has reported the influence of IBW on vancomycin volume of distribution in critically ill patients under CI. Indeed, vancomycin dosing based on total body weight remains widespread in clinical guidelines and routine practice. We observed large intra-individual variability in total body weight in our study population. Those can be explained by the administration of IV fluids, inotropic agents, and diuretics that cause changes in body water. We assume that TBW intra-individual variability may be larger than that of vancomycin V1 and CL and that a fixed estimate of body size such as IBW may be more suited as a covariate. In another study, vancomycin dosing based on IBW was reported to be a better predictor of vancomycin exposure than TBW in obese patients [29].

Vancomycin clearance was influenced by renal function (CR_CL_ based on IBW) and CRRT, as expected considering the renal transport of vancomycin… Darren et al. reported a great variability of vancomycin pharmacokinetics in patients receiving CRRT [30]. It has been shown that vancomycin CL may vary with the CRRT technique and its intensity [31]. This is why we considered CRRT_EFR_ as a quantitative descriptor of vancomycin CL clearance instead of setting CRRT as a binary variable. Overall, the use of CRRT_EFR_ and CG_IBW_ resulted in the best estimation of vancomycin clearance in our cohort.

Previous studies recommended maintenance dosing regimens of vancomycin ranging from 20 to 35 mg/kg (TBW) to achieve target plateau concentration of 20–25 mg/L [18,23]. Dosage regimens based on our model to achieve similar exposure appear to be lower, ranging from 12.5 to 30 mg/kg of IBW. This dosing approach aims at minimizing both under- and overexposure. An alternative dosing approach aiming at minimizing underexposure only is also presented (Table 4) and resulted in higher dosages ranging from 20 to 35 mg/kg of IBW We calculated the equivalent dosages in mg/kg of TBW to be 16.6 to 28.8 mg/kg, those being in agreement with previous reports. Of note, many previous studies consider vancomycin intermittent infusion and a target trough of 15–20 mg/L. It has been shown that trough concentration of vancomycin poorly correlated with daily AUC and that many patients can achieve daily AUC > 400 with trough concentration < 15 mg/L [32]. Thus, AUC-based monitoring should be associated with dosage requirements lower than previously recommended when vancomycin is administered by intermittent infusion.

This study has several limitations. First, it was performed in routine clinical conditions and some errors may have occurred in blood sampling and data recording. Second, blood sampling was sparse, which may alter the precision of estimation for some PK parameters, such as V1 and Q. Finally, information on other drugs co-administered with vancomycin was not recorded.

Overall, our model performed well with low bias and acceptable precision. External validation yielded similar results, suggesting robust application in critically ill patients. Continuous vancomycin infusion appear safer than intermittent infusion [33,34]. Our model-based dosing regimens may be helpful for initial dosing. However, due to high intra and inter-individual variability, the expected proportion of patients reaching the optimal exposure target remains limited. TDM and model-based precision dosing remains necessary to maximize efficacy while minimizing the risk of AKI as previously shown [12].

## 4. Materials and Methods

### 4.1. Data Collection

This study was carried out in patients hospitalized in two ICU centers during two different periods.

Data of the first population of patients (learning dataset) were collected between December 2013 and April 2015 in center 1 (Croix-Rousse Hospital, University Hospitals of Lyon, France). Inclusion criteria were as follows: age ≥ 18 years, hospitalization in ICU, administration of continuous IV vancomycin, vancomycin therapy initiated in the ICU unit, vancomycin TDM with at least one measured vancomycin concentration, and hemodynamic monitoring with the Picco^®^ device [35]. The latter criterion was necessary to investigate the relationships between some hemodynamic parameters and vancomycin PK parameters. Patients that had vancomycin treatment for less than 24 h, patients without vancomycin TDM, patients with rare comorbidities influencing vancomycin pharmacokinetics such as myeloma, cystic fibrosis or burn injury on more than 20% of the body surface were excluded. The invasive hemodynamic monitoring justified the approval of an ethics committee for this part of the study. This approval was granted (CPP Sud-Est 2, IRB number 00009118) and the study was registered on clinical trials website.

Data of the second population of patients (validation dataset) were retrospectively collected from February 2019 to September 2020 in center 2 (Centre Hospitalier Lyon Sud, University Hospitals of Lyon, France) as part of vancomycin routine TDM. As we performed a retrospective analysis of anonymized data collected in routine care, patients’ consent and ethics approval were not required for this part, as stated in French regulations for clinical research [36].

In both populations, TDM was performed as part of routine patient care, in accordance with guidelines [37]. In most patients, TDM was performed on several occasions to control drug exposure and minimize the risk of adverse events throughout therapy. For each patient, vancomycin samples were collected each morning and sampling times were recorded precisely.

Vancomycin assays were performed by an immunoturbidimetry method on an Abbott Architect C8000 analyzer. In plasma, the lower limit of quantification was 1.1 μg/mL, and the linear range of the assay was 1.1 to 100 μg/mL. In the population used for model building, several variables were collected from the computerized patient files, including anthropometric measurements such as age, sex, total body weight (TBW) measured every day, height, body temperature, SOFA, BMI, and the presence of septic shock at admission time as defined by the Sepsis 3 guidelines [38]. Alternative body size metrics such as ideal body weight (IBW, using Devine formula) and adjusted body weight (AjBW) were derived from TBW [39,40]. Biological variables were also collected, including serum creatinine, serum protein, serum albumin, and creatinine clearance (CR_CL_) estimated by the Cockcroft–Gault equation with total (CG_TBW_), ideal (CG_IBW_), and adjusted body weight (CG_AjBW_). If the patient had anuria or was undergoing CRRT, the value of CR_CL_ was set to 0 mL/min. The use of CRRT was also collected, as well as the CRRT effluent flow rate (CRRT_EFR_) calculated according to the CRRT technique. It is important to note that all patients were administered continuous RRT. As patients were under invasive hemodynamic monitoring PiCCO^®^ (Pulsion Medical Systems, Feldkirchen, Germany), pulmonary vascular permeability index (PVPI), extravascular lung water index (EVLWI), and cardiac index were also collected. Finally, information regarding vancomycin therapeutic drug monitoring (TDM) including dosing history and administration times were collected.

Data used in routine vancomycin TDM were collected in another group of critically ill patients in another center of the University Hospitals of Lyon from February 2019 to September 2020.

### 4.2. Pharmacokinetic Modeling

Population pharmacokinetic (PPK) modeling was performed using Monolix software (2020R1 Version, Lixoft, Antony, France). We used a nonlinear mixed effect modeling approach and the Stochastic Approximation of Expectation maximization algorithm (SAEM) implemented in Monolix. We assumed a log normal distribution of PK parameters to describe inter individual variability (IIV). Continuous covariates were introduced into the model as follows (1):

(1) $$X=X_{pop}\;{\left(\frac{COV}{COV_{median}}\right)}^{\alpha }$$

where *X* is the parameter value (e.g., clearance, volume), *X_pop_* is the population value of *X*, *COV* is the covariate value normalized by the median value in the population (*COV_median_*), and α is the coefficient of the power relationship.

The model for categorical covariate was as follows (2):

(2) $$X=\;X_{pop}{\left(\beta_{COV}\right)}^{COV}\;$$

where *X* is the pharmacokinetic parameter, *COV* is the binary covariate value (0 or 1), *X_pop_* is the population value of *X* when *COV* = 0, and *β_COV_* is the coefficient of the covariate effect.

All covariates collected were tested to study their influence on PK parameters using a stepwise procedure, based on changes observed in the objective function value (OFV) computed by Monolix. OFV was assumed to follow a chi-squared distribution. P-values of 0.05 and 0.01 were considered statistically significant for forward selection and backward deletion of covariates, respectively. The best structural error and covariate model was identified based on several classical criteria and diagnostics including the objective function value, parameter value along with their relative standard error, plots of predicted vs observed concentration and residuals, and simulation-based diagnostics with prediction-corrected visual predictive checks (VPC).

External validation was performed with the validation dataset. Predictive performance was assessed by comparing observed vs predicted concentration plots and computation of bias (mean error of prediction), mean absolute error (MAE) and root mean squared error (RMSE) in both datasets. Details about validation criteria in population pharmacokinetics have been presented elsewhere [41,42].

### 4.3. Dosing Simulations

The final model was then implemented in Simulx (2020R1 Version, Lixoft, Antony, France) to perform simulations of various loading and maintenance dosing regimens.

Because CR_CL_ influenced vancomycin clearance in the final model, simulations were performed for various stages of CR_CL_ ranging from 10 to 150 mL/min, with a 10 mL/min increment. In addition, two values of CRRT_EFR_ of 30 and 20 mL/min were considered for dosing in patients under CRRT. As IBW was found to influence vancomycin volume of distribution, weight-based dosing was used in all simulations. For each stage of renal function, dosages ranging from 5 to 40 mg/kg of IBW were simulated, with a 2.5 mg/kg increment.

For each set of simulations, 500 virtual patients were simulated and their vancomycin PK profiles were estimated. Each simulation was run 20 times, in order to account for variability in random sampling. The endpoint considered was the AUC24–48 h on the second day of therapy, because the loading dose resulted in higher vancomycin exposure on the first day. The AUC24–48 h target on day 2 was based on 2020 IDSA guidelines [14]. We assume a putative MIC of 1 mg/L for probabilistic sepsis therapy. The efficacy target was AUC24–48 h > 400 mg·h/L, while the safety target was set at AUC24–48 h < 600 mg·h/L. The optimal dosage was defined as the one that maximized the proportion of simulated patients with AUC24–48 h between 400 and 600 mg·h/L, which is supposed to maximize both efficacy and safety.

Considering that an alternative goal of initial therapy may be to maximize the effect in some patients with severe infection, a second set of simulations was performed to identify the dosage that would minimize underexposure, so maximizing the proportion of AUC24–48 h > 400 mg·h/L. A proportion ≥ 90% was considered as acceptable.

## 5. Conclusions

This is the largest population pharmacokinetic study of vancomycin administered by CI in critically ill patients. Our final model provided good predictive performance in external validation. Our results support IBW as a covariate, influencing vancomycin PK and dosing. Based on dosing simulations, we suggest a loading dose of 27.5 mg per kg of IBW in all patients. Maintenance dose should be adjusted to renal function or CRRT intensity. Those regimens should optimize the achievement of AUC target of 400–600 mg·h/L over the first two days of therapy. TDM remains necessary for individualization throughout therapy in those unstable patients.

## Figures and Tables

**Table 1:** Baseline characteristics of patients.

Demographic	Learning Dataset	Validation Dataset
Number of Men/Women	57/21	48/36
Age (years)	68.9 ± 12.3	58.9 ± 15.3
Weight (kg)	77.9 ± 20.4	81.4 ± 18.9
IBW (kg)	63.5 ± 9	61.4 ± 8.8
Height (cm)	168.7 ± 8.5	168 ± 8.3
BMI (kg/m^2^)	27.6 ± 7.7	28 ± 7.9
SOFA	11 ± 4	NA
Temperature (°C)	37.8 ± 1.1	NA
Septic shock (%)	83.3	NA
IGS-II	55.9 ± 17.3	NA
CRRT (%)	28.2	4.8
**Blood tests**		
Serum creatinine (µmol·L^−1^)	139.3 ± 75.7	149 ± 123
Serum protein (g·L^−1^)	60 ± 11.3	NA
Serum albumin (g·L^−1^)	23.1 ± 5.9	NA
Lactate (mmol·L^−1^)	3.8 ± 3.4	NA
Procalcitonin (ng·mL^−1^)	2.4 [0.8–6.8]	NA
CR_CL_ (mL·min^−1^)	50.4 ± 29	55.2 ± 37.4
**Dialysis parameters**		
CRRT_EFR_ (mL·min^−1^)	35.6 ± 18.7	28.7 ± 6.5
**PiCCO variables**		
EVLWI (mL·kg^−1^)	9.2 ± 5.5	NA
PVPI	1.9 ± 1	NA
Cardiac index (µmol·L^−1^)	3.0 ± 1.5	NA
**Vancomycin TDM**		
Loading dose (mg/kg)	22.7 ± 7.5	22 ± 5.8
Mean maintenance dose (mg/kg)	28.6 ± 9.4	25.2 ± 7.6
AUC24–48 h (mg·h/L^−1^)	530 ± 160	515 ± 341
AUC24–48 h > 400 (%)	83.3	70.5
400 < AUC24–48 h < 600 (%)	59	25.7
AUC24–48 h > 600 (%)	24.3	44.8

IBW, ideal body weight; BMI, body mass index; SOFA, sequential organ failure assessment; CR_CL_, creatinine clearance; CRRT, continuous renal replacement therapy; CRRT_EFR_, CRRT effluent flow rate; ELWVI, extravascular lung water index; PVPI, pulmonary vascular permeability index; NA, not available. Data are described as mean ± SD or median (IQR) unless otherwise stated.

**Table 2:** Estimates of pharmacokinetic parameters of the final model.

	Value	RSE (%)
**Fixed effects**		
**CL_pop_ (L/h)**	0.79	12.7
**V1_pop_ (L)**	27.3	45.1
**Q_pop_ (L/h)**	6.08	41.8
**V2_pop_ (L)**	61.3	9.7
**α**	1.88	67.1
**Random effects (standard deviation)**		
**ω_CL_**	0.76	11.1
**ω_V_**	0.61	51.1
**ω_Q_**	0.49	33.8
**ω_V2_**	0.48	16.5
**Residual error model**		
**b**	0.13	6.3
**X_pop_ is the population value (fixed effect) of the corresponding parameter X (clearance or volume) and ω_X_ its corresponding random effect. The final equations of individual parameters were as follows:** $CL_{i}=CL_{0\;}*\;e^{\eta_{CL_{i}}}\;with\;\eta_{CL_{i}}\sim N\left(0,\omega_{CL}\right)\;and\;\left\{\begin{array}{c} \;CL_{0}=CL_{pop}e^{{\left(\frac{CR_{CL_{i}}}{41.4}\right)}^{0.5}},\;if\;CRRT=0\\ \;CL_{0}=CL_{pop}e^{{\left(\frac{CRRT_{EFR_{i}}}{20}\right)}^{0.69}},\;if\;CRRT=1 \end{array}\right.$ $V_{1_{i}}=V_{1pop}e^{{\left(\frac{IBW}{64.1}\right)}^{\alpha }}*\;e^{\eta_{vi}}\;with\;\eta_{V_{1i}i}\sim N\left(0,\omega_{v}\right)$ $Q_{i}=Q_{pop}\;*\;e^{\eta_{Qi}}\;with\;\eta_{Q_{i}}\sim N\left(0,\omega_{Q}\right)$ $V_{2_{i}}=V_{pop}\;*\;e^{\eta_{V_{2i}}}\;with\;\eta_{V_{2i}}\sim N\left(0,\omega_{V_{2}}\right)$		

**Table 3:** Optimal maintenance dose based on simulations with the final model.

	Optimal Maintenance Dose (mg/kg/24 h) ^a^	AUC_24–48_ (mg·h/L) ^b^	PTA for AUC_24–48_ > 400 (mg·h/L) ^c^	400 < AUC_24–48_ < 600 (mg·h/L) ^c^	PTA for AUC_24–48_ > 600 (mg·h/L) ^c^
CR_CL_ (mL/min)					
150	30	493 [330–644]	0.80	0.64	0.16
140	30	506 [335–666]	0.83	0.62	0.21
130	27.5	483 [316–643]	0.77	0.63	0.14
120	27.5	499 [324–668]	0.80	0.61	0.19
110	25	475 [303–646]	0.74	0.60	0.14
100	25	492 [313–674]	0.77	0.58	0.19
90	25	508 [320–704]	0.80	0.55	0.25
80	22.5	484 [298–684]	0.75	0.56	0.19
70	22.5	503 [307–720]	0.78	0.55	0.23
60	20	486 [287–706]	0.74	0.53	0.21
50	20	506 [295–751]	0.77	0.50	0.27
40	17.5	472 [273–707]	0.69	0.50	0.19
30	17.5	498 [283–761]	0.74	0.47	0.27
20	15	480 [265–767]	0.69	0.44	0.25
10	12.5	470 [245–776]	0.66	0.42	0.24
CRRT Effluent Flow Rate (mL/min)					
30	20	494 [295–723]	0.74	0.5	0.24
20	17.5	480 [277–728]	0.71	0.49	0.22

^a^ All vancomycin maintenance dosages were simulated after the same loading dose of 27.5 mg/kg (IBW) administered over 2 h. Maintenance dose are expressed in mg/kg of ideal body weight over a 24 h infusion. ^b^ AUC_24–48_ corresponds to the AUC estimated during the second day of therapy with results reported as median (95% tolerance interval). ^c^ PTA are given as proportions.

**Table 4:** PK/PD simulation for probability of target attainment > 90%.

	Optimal Maintenance Dose (mg/kg/24 h) ^a^	AUC_24–48_ (mg·h/L) ^b^	PTA forAUC_24–48_ > 400 (mg·h/L) ^c^	PTA for AUC_24–48_ > 600 (mg·h/L) ^c^
CR_CL_ (mL/min)				
150	35	557 [370–728]	0.91	0.39
140	32.5	538 [355–708]	0.89	0.32
130	32.5	552 [362–735]	0.90	0.37
120	32.5	566 [368–758]	0.91	0.43
110	30	547 [351–742]	0.88	0.36
100	30	563 [358–771]	0.89	0.42
90	30	580 [365–802]	0.91	0.48
80	27.5	562 [349–788]	0.89	0.42
70	27.5	582 [358–828]	0.90	0.48
60	27.5	604 [365–874]	0.91	0.54
50	25	588 [352–869]	0.89	0.50
40	25	598 [355–899]	0.89	0.52
30	25	626 [368–968]	0.91	0.59
20	22.5	614 [348–976]	0.89	0.56
10	20	615 [331–1013]	0.89	0.55
CRRT Effluent Flow Rate (mL/min)				
30	27.5	613 [371–899]	0.91	0.56
20	25	610 [359–911]	0.90	0.54

^a^ All vancomycin dosages were simulated after the same loading dose of 27.5 mg/kg (IBW) administered over 2 h. Maintenance dose are expressed in mg/kg of ideal body weight over a 24 h infusion. ^b^ AUC_24–48_ correspond to the AUC estimated during the second day of therapy, with results reported as median (95% tolerance interval). ^c^ PTA are given as proportions.

## Data Availability

The data presented in this study are available upon request.
